# *BioXTAS RAW 2*: new developments for a free open-source program for small-angle scattering data reduction and analysis

**DOI:** 10.1107/S1600576723011019

**Published:** 2024-02-01

**Authors:** Jesse B. Hopkins

**Affiliations:** a The Biophysics Collaborative Access Team (BioCAT), Department of Physics, Illinois Institute of Technology, Chicago, IL 60616, USA; Argonne National Laboratory, USA

**Keywords:** small-angle scattering, data analysis, biological macromolecules, *BioXTAS RAW 2*, computer programs

## Abstract

*BioXTAS RAW* is a free open-source program for reduction, analysis and modelling of biological small-angle scattering data. Here, the new developments in *RAW* version 2 are described. These include improved data reduction using *pyFAI*; updated automated Guinier fitting and *D*_max_ finding algorithms; automated series (*e.g.* size-exclusion chromatography coupled small-angle X-ray scattering or SEC-SAXS) buffer- and sample-region finding algorithms; linear and integral baseline correction for series; deconvolution of series data using regularized alternating least squares (*REGALS*); creation of electron-density reconstructions using electron density via solution scattering (*DENSS*); a comparison window showing residuals, ratios and statistical comparisons between profiles; and generation of PDF reports with summary plots and tables for all analysis. Furthermore, there is now a *RAW* API, which can be used without the graphical user interface (GUI), providing full access to all of the functionality found in the GUI. In addition to these new capabilities, *RAW* has undergone significant technical updates, such as adding Python 3 compatibility, and has entirely new documentation available both online and in the program.

## Abstract 2

*BioXTAS RAW* is a free open-source program for reduction, analysis and modelling of small-angle scattering data. This article describes the new and improved features in *RAW* version 2, including new tools for liquid-chromatography coupled data processing, advanced reporting capabilities and a new API.

## 1. Introduction

Small-angle solution scattering (SAS) of both X-rays (SAXS) and neutrons (SANS) is a popular structural technique for studying biological macromolecules. SAS provides information on the solution state of macromolecules and complexes, including, but not limited to, size and molecular weight (MW), flexibility, degree of folding, and overall shape (Trewhella, 2022 ▸; Da Vela & Svergun, 2020 ▸; Brosey & Tainer, 2019 ▸; Meisburger *et al.*, 2017 ▸; Jacques & Trewhella, 2010 ▸; Svergun & Koch, 2003 ▸). The growing popularity of SAS as part of the structural biology toolbox has many contributing factors: expanding data-collection capabilities for more challenging systems, including the increasing number of hyphenated techniques like size-exclusion or ion-exchange chromatography coupled to SAXS (SEC-SAXS and IEC-SAXS) (Graewert & Svergun, 2022 ▸; Pérez *et al.*, 2022 ▸); increasing automation of data collection and analysis to make the technique more accessible for new users (Tully *et al.*, 2023 ▸; Rosenberg *et al.*, 2022 ▸; Lazo *et al.*, 2021 ▸); and an increasing awareness that SAS is highly complementary to other structural and biophysical techniques such as X-ray crystallography (MX), nuclear magnetic resonance, cryo-electron microscopy (cryo-EM), and multi-angle (also called static) and dynamic light scattering (Trewhella, 2022 ▸; Brosey & Tainer, 2019 ▸; Grishaev, 2017 ▸). Additionally, SAS has proven an invaluable tool for studying intrinsically disordered proteins and liquid–liquid phase separating systems, which are not readily amenable to common high-resolution structural techniques such as MX and cryo-EM (Lenton *et al.*, 2022 ▸; Martin, Hopkins *et al.*, 2021 ▸; Martin *et al.*, 2020 ▸; Sagar *et al.*, 2020 ▸; Riback *et al.*, 2017 ▸; Kikhney & Svergun, 2015 ▸).

Providing a detailed review of SAS techniques and data analysis is beyond the scope of this article (interested readers are directed to any number of excellent reviews, such as those cited previously). Briefly, data are collected as two-dimensional detector images, which are then radially averaged into one-dimensional scattering profiles. Both the sample in solution and the solution by itself are measured, and the solution blank (‘buffer’) measurement is subtracted from the sample measurement to create a subtracted scattering profile (Skou *et al.*, 2014 ▸). Sometimes, more advanced deconvolution methods must be used to determine the scattering profiles of overlapping components in the dataset (Tully *et al.*, 2021 ▸). Model-free analysis of the subtracted profile is then carried out – including a Guinier fit, calculation of MW, creation of the pair-distance distribution [*P*(*r*)] function by indirect Fourier transform (IFT), and creation of specific plots like the Kratky and Porod plots – to provide initial information about the sample. Depending on the system, more advanced analysis can be carried out, including *ab initio* reconstruction of low-resolution models, fitting high-resolution models to the data, and modelling flexibility or polydispersity in the solution (Skou *et al.*, 2014 ▸; Dyer *et al.*, 2014 ▸).

A wide variety of software tools have been developed by the SAS community to carry out data reduction, analysis and modelling, and we can provide only a non-exhaustive overview. The most popular software remains the *ATSAS* package (both desktop and web versions) (Manalastas-Cantos *et al.*, 2021 ▸), which provides everything from initial reduction through to advanced analysis and modelling, such as rigid-body modelling with *SASREF* (Petoukhov & Svergun, 2005 ▸) and bead-model reconstructions with *DAMMIF* (Franke & Svergun, 2009 ▸). *ScÅtter* is another general-purpose analysis package (https://github.com/rambor/scatterIV). Other data-reduction options include *pyFAI*, *DPDAK* and *FIT2D* (Kieffer *et al.*, 2020 ▸; Ashiotis *et al.*, 2015 ▸; Benecke *et al.*, 2014 ▸; Hammersley *et al.*, 1996 ▸; Hammersley, 2016 ▸), along with beamline-specific processing pipelines (which may rely on one of the previous software packages to do the reduction) (Tully *et al.*, 2023 ▸; Thureau *et al.*, 2021 ▸; Cowieson *et al.*, 2020 ▸) and software provided by laboratory X-ray source companies. Numerous tools are available for more specific types of analysis including (but not limited to) *SASSIE*, *FoXS* (and *FoXSDock* and *MultiFoXS*), *WAXSiS*, *DADIMODO* and *BilboMD* (all available as web servers) for testing and/or modelling high-resolution data against SAS profiles (Curtis *et al.*, 2012 ▸; Schneidman-Duhovny *et al.*, 2016 ▸; Knight & Hub, 2015 ▸; Evrard *et al.*, 2011 ▸; Pelikan *et al.*, 2009 ▸); the electron density via solution scattering algorithm (*DENSS*) and *Memprot* for low-resolution electron density or bead-model reconstructions from SAS profiles (Grant, 2018 ▸; Pérez & Koutsioubas, 2015 ▸); and regularized alternating least squares (*REGALS*) and *US-SOMO* for deconvolution of data (Meisburger *et al.*, 2021 ▸; Brookes *et al.*, 2016 ▸). Many other programs exist and readers are encouraged to carry out a literature search or consult with experts to find the best options for their particular analysis.

The *BioXTAS RAW* program is most similar to *Primus* (*ATSAS* package) (Konarev *et al.*, 2003 ▸) and *ScÅtter*, in that it provides a user-friendly graphical user interface (GUI) with both model-independent and model-dependent analysis, though unlike those programs *RAW* allows users to start with and reduce images to scattering profiles. Our focus in making *RAW* is to provide an open-source, free, easy-to-learn and easy-to-use program capable of doing the majority of what users need, including data reduction, producing subtracted profiles, carrying out standard analysis such as Guinier fits and generating *P*(*r*) functions, and performing some more advanced analysis such as shape reconstructions and deconvolution of overlapping chromatography coupled SAS data. *RAW* also provides a home for some advanced analysis techniques by directly incorporating (with permission, and often assistance, from the authors) techniques developed by others. This makes these techniques easier to use (*e.g.* by providing a GUI and documentation) and expands the number of users able to take advantage of these developments. Finally, *RAW* also incorporates some popular tools from the *ATSAS* package (separate installation required) for a more unified experience in *RAW*. Because one of our goals is for *RAW* to be an open-source toolbox for SAS, we have been working to provide open-source alternatives to all of the *ATSAS* tools we incorporate, and with the exception of theoretical profile calculation with *CRYSOL* this is now the case. Thus, even without *ATSAS* available, *RAW* provides a full range of reduction and analysis techniques for its users.

## 2. Program overview

**Figure 1:**
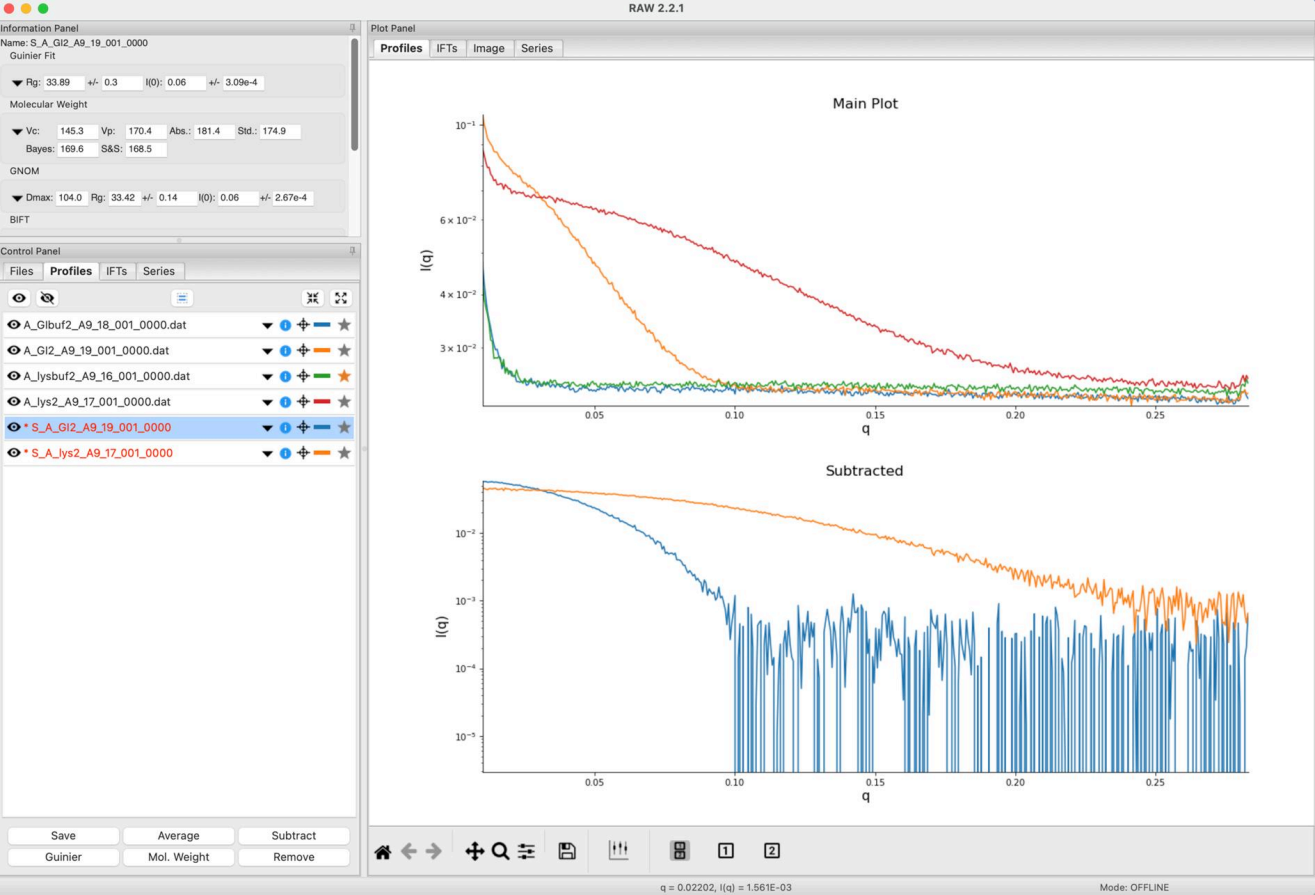
The main *RAW* window showing data for two batch-mode protein datasets. The top plot shows the unsubtracted protein and buffer profiles while the bottom plot shows the subtracted protein profiles.

Many of the basics of the *RAW* program have been previously described (Hopkins *et al.*, 2017 ▸; Nielsen *et al.*, 2009 ▸), so this article focuses on updates to the program since the last publication. Here, we provide a brief summary of the program and its capabilities for readers not familiar with *RAW*. The main window of *RAW* is shown in Fig. 1 ▸.

*RAW* is a GUI-based, free and open-source Python program for reduction and analysis of both SAXS and SANS data. The software is designed for biological SAS data. It is available on Windows, macOS (and OS X) and Linux. With *RAW*, users can carry out analysis starting with detector images and go all the way through to three-dimensional reconstructions. They can mask, calibrate and radially average X-ray images into one-dimensional scattering profiles as *I*(*q*) versus *q*, where 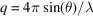 is the usual scattering vector magnitude, with 2θ being the scattering angle and λ being the wavelength (*RAW* is ambivalent to the units of *q* and users must label plots with the appropriate units, typically Å^−1^ or nm^−1^). *RAW* provides users with basic manipulation of scattering profiles including averaging, subtraction, rebinning, scaling intensity and *q*, and trimming the *q* range. Once a subtracted scattering profile is available, generated either in *RAW* or in another program, there are a host of analysis and visualization tools available. Users can plot on linear and logarithmic axes in both intensity and *q*; generate Guinier, Kratky and Porod plots; and create normalized and dimensionless Kratky plots (Durand *et al.*, 2010 ▸). Users can perform Guinier fits to yield *R*_g_ (radius of gyration) and *I*(0). MW analysis is available to users by a number of methods: using a reference standard (Mylonas & Svergun, 2007 ▸), absolute scale calibration (Orthaber *et al.*, 2000 ▸), the correlation volume (*V*_c_) method (Rambo & Tainer, 2013 ▸), the adjusted Porod volume (*V*_p_) method (Fischer *et al.*, 2010 ▸; Piiadov *et al.*, 2019 ▸), the *ATSAS* *Shape&Size* method (Franke *et al.*, 2018 ▸) and the *ATSAS* Bayesian inference method (Hajizadeh *et al.*, 2018 ▸). Users can generate *P*(*r*) functions using IFTs via either the *ATSAS GNOM* software (Svergun, 1992 ▸) or the Bayesian IFT (BIFT) method (Hansen, 2000 ▸).

After a *P*(*r*) function is made available, generated either in *RAW* or in another program, *RAW* users can carry out an ambiguity assessment of the three-dimensional reconstructions using the *ATSAS AMBIMETER* program (Petoukhov & Svergun, 2015 ▸). Users can then carry out three-dimensional reconstructions, averaging, clustering, refinement and alignment to high-resolution structures within *RAW* using the *ATSAS* approach for bead models including *DAMMIF*, *DAMMIN*, *DAMAVER*, *DAMCLUST*, *SUPCOMB/CIFSUP* and *SASRES* (Franke & Svergun, 2009 ▸; Svergun, 1999 ▸; Volkov & Svergun, 2003 ▸; Petoukhov *et al.*, 2012 ▸; Kozin & Svergun, 2001 ▸; Tuukkanen *et al.*, 2016 ▸). Alternatively, users can reconstruct electron density via the same steps minus clustering using *DENSS* (Grant, 2018 ▸).

*RAW* provides users with basic and advanced capabilities for dealing with liquid-chromatography coupled SAS (LC-SAS) data and other sequentially sampled data. Users can load data as a series, and for standard SEC-SAS a buffer region can be automatically selected and then subtracted from the dataset. *R*_g_ and MW are calculated across the elution peaks. The peak region of interest can be automatically selected and the subtracted one-dimensional profile from that peak region can be generated for further analysis. *RAW* also provides users with the ability to carry out linear and integral baseline corrections to the data to account for various forms of background drift (Brookes *et al.*, 2013 ▸, 2016 ▸). For more complicated series datasets, two deconvolution methods are available to users. Evolving factor analysis (EFA) deconvolution focuses mostly on SEC-SAS data or other data where components obey a first-in/first-out principle (Meisburger *et al.*, 2016 ▸), whereas *REGALS* provides a more general deconvolution approach that can apply additional constraints to the data such as smoothness or a real-space restraint with a *P*(*r*) function (Meisburger *et al.*, 2021 ▸). This makes *REGALS* more amenable to complicated datasets including titration series, time-resolved measurements and IEC-SAS.

In addition to the GUI version of the program, users can install *RAW* as a Python package, giving access to the new *RAW* API, which they can use for custom Python scripts, for *Jupyter Notebooks* (https://jupyter.org/), and in other programs (such as beamline processing pipelines) where they want to use the tools available in *RAW* in a more automated or scriptable fashion.

## 3. Availability

The *RAW* source code is GPL-3.0 licenced and available on GitHub: https://github.com/jbhopkins/bioxtasraw. Official versioned releases of *RAW* are available for download on Sourceforge: https://sourceforge.net/projects/bioxtasraw/. Older releases of *RAW* can also be found on Sourceforge. These official versioned releases include the source code associated with that version as a zip file and prebuilt installers for Windows, macOS (both x86_64 and arm64 native) and Linux (a deb installer). Both the source code and installers are freely available for anyone to download, use and share. *RAW* is also available through SBGrid. The *RAW* documentation is hosted on Read the Docs: https://bioxtas-raw.readthedocs.io/en/latest/.

## 4. Improvements to previously described features

### 4.1. Updates to series data processing

**Figure 2:**
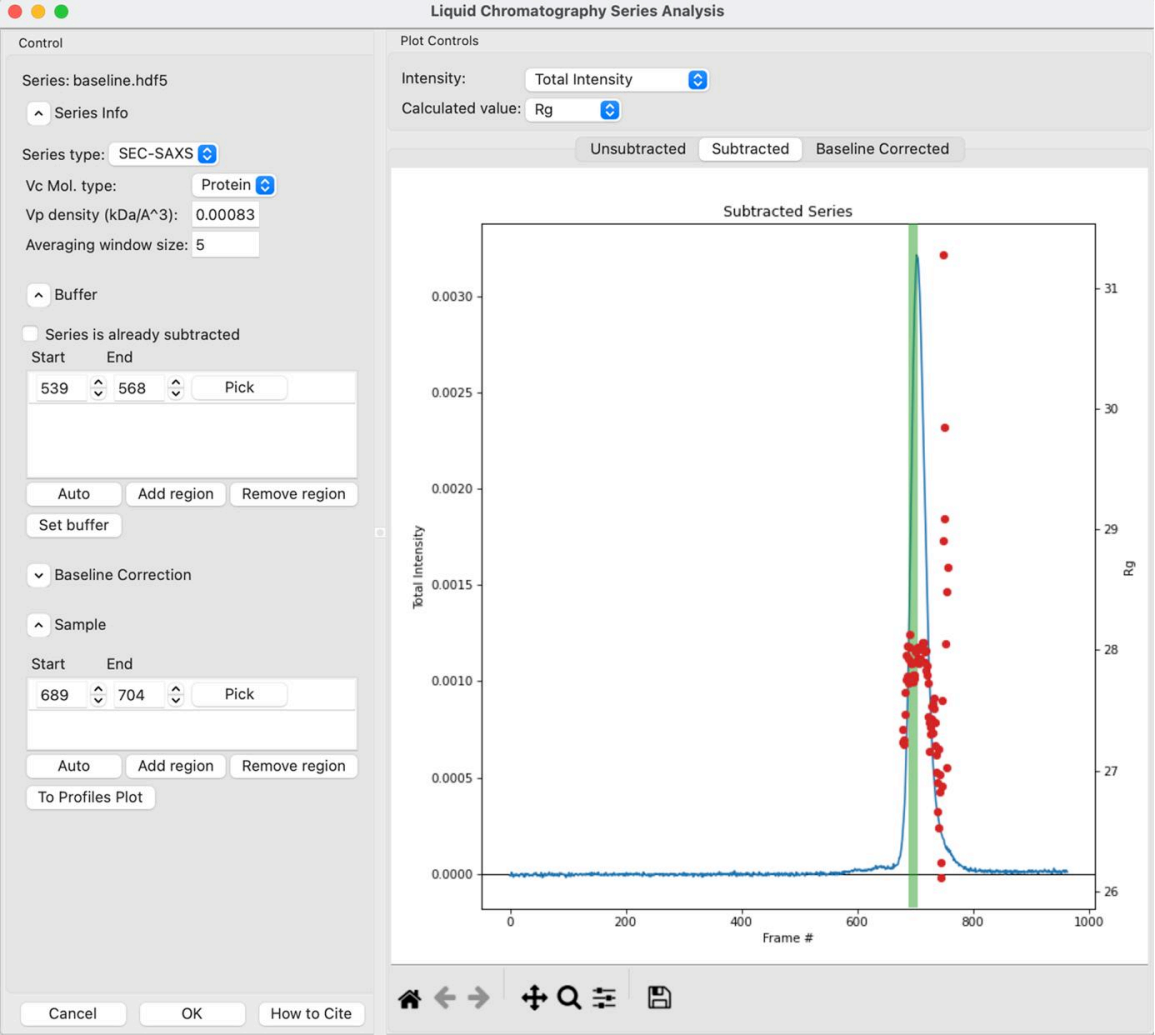
The new LC Series Analysis window in *RAW*, which allows automated and manual selection of buffer and sample ranges and baseline correction and plots the *R*_g_ and MW values across the elution peak.

The part of *RAW* that has undergone the most significant update since the last report on the program is the series processing capabilities. The core of the new features is a new LC analysis module (Fig. 2 ▸) that provides buffer subtraction, baseline correction and sample-range averaging all in one place. Additionally, there is a new deconvolution method for series data, *REGALS*, which is described in Section 5.3. One relatively minor but quite useful new feature is that users can now scale profile intensity and adjust the *q* range of profiles for all the profiles in a series at once.

Before discussing the new features, it is useful to understand the general LC analysis workflow in *RAW*. First, a user will load in a series, which could be from .dat files, image files or a previously saved *RAW* series file. The user then opens the LC analysis window. If any previous analysis has been done and saved, it is then displayed; if not, the user proceeds as described below.

First, *RAW* displays a plot of the total intensity in each profile versus frame number. The user then selects a buffer range (either automatically, manually or a combination of the two), which can be multiple disconnected regions of the data (*e.g.* pre- and post-peak buffer can be averaged). Once the user has selected a buffer range, *RAW* averages the buffer profiles and then subtracts this average buffer from every profile in the dataset. *RAW* then attempts to calculate *R*_g_ and MW for each profile in the dataset, using a sliding average window as previously described (Hopkins *et al.*, 2017 ▸). The user sees a new plot of *R*_g_ or MW (user selectable) and the subtracted total intensity versus frame number. The user may optionally then perform a baseline correction (see Section 4.1.3). If they do, the correction is applied, *R*_g_ and MW are recalculated for the baseline-corrected data, and the baseline-corrected total intensity versus frame number is plotted along with the new *R*_g_ and MW values on a new plot. The baseline itself is also plotted with the subtracted (but not baseline corrected) intensity for the user to examine.

**Figure 3:**
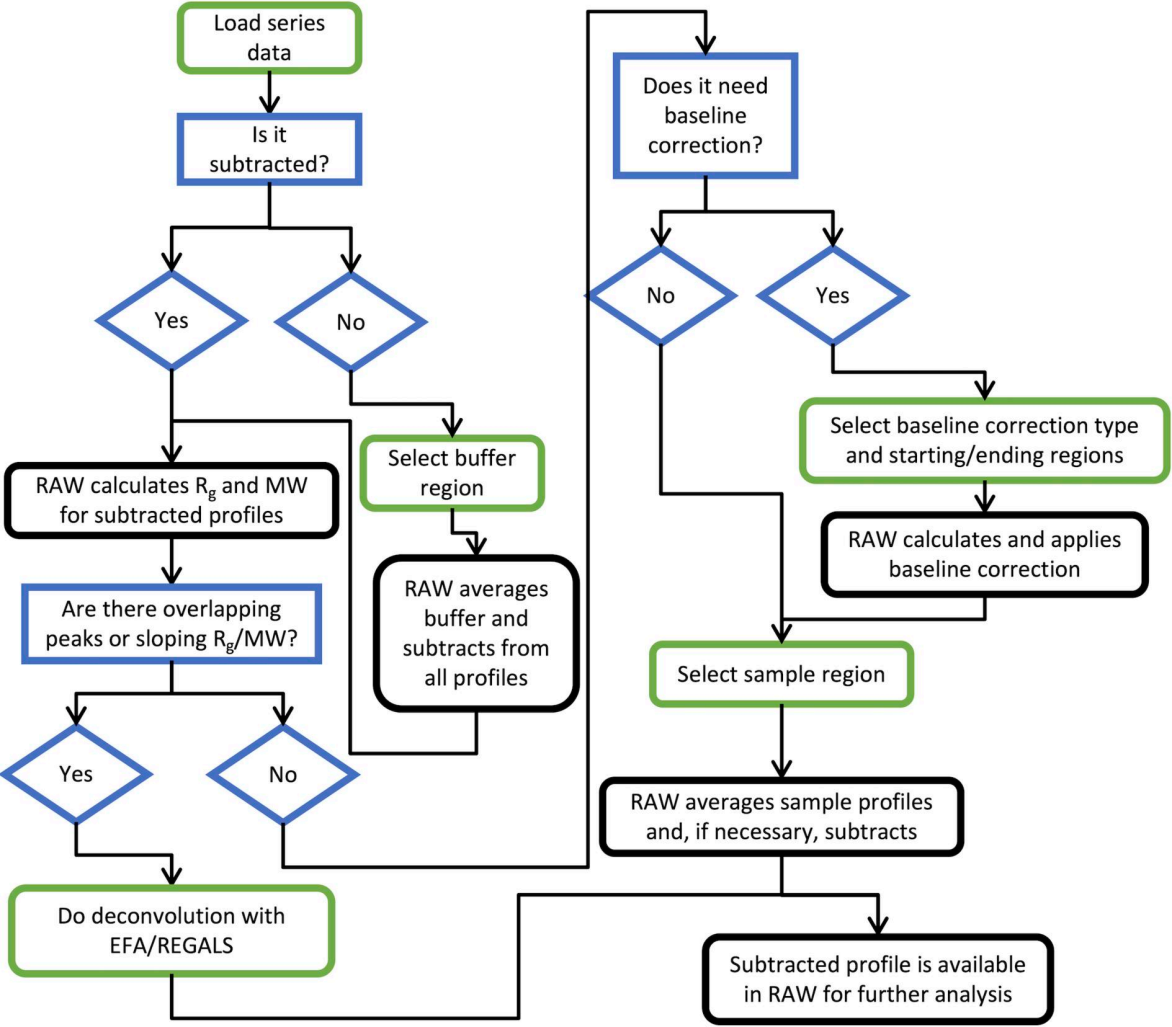
A decision tree for standard SEC-SAS analysis using *RAW*. Green-edged boxes (rounded corners) indicate user actions (though many can be either manual or automated, such as picking a buffer region). Blue-edged boxes (square corners) and diamonds indicate decisions, and black-edged boxes (rounded corners) indicate processing done by *RAW*.

The user next selects a sample range (either automatically, manually or a combination of the two), which can consist of multiple disconnected regions of the data, for further processing. Once the sample range is set, the original unsubtracted data are averaged across the selected sample range and the averaged buffer profile is subtracted. This final subtracted profile is sent to the Profiles plot where the user may continue analysis. This final subtraction step avoids averaging subtracted data where the same buffer profile has been used for subtraction of each profile, which would introduce correlations in the uncertainty values. The user can also work with previously subtracted data, skipping the buffer-subtraction step. A more complete decision tree for standard SEC-SAS analysis is shown in Fig. 3 ▸.

The selected buffer ranges, baseline-correction parameters and sample ranges are saved with the series data in *RAW*. If the LC analysis window is reopened, all the parameters are repopulated and can easily be tweaked by the user. These parameters are also saved with the profiles in the .hdf5 file when series data are saved, so data loaded into *RAW* from file have access to the previous analysis parameters.

In addition to the total integrated intensity, *RAW* can also display the mean intensity, the total intensity in a user-defined *q* range and the intensity at a specific *q* value. Different approaches to displaying the intensity have different use cases. The total intensity is the default choice in *RAW* because it provides a better view of possible issues with the dataset, such as being able to see capillary fouling or baseline drift at low or high *q*. Intensity in a specific *q* range (such as intensity from 0.02 to 0.2 Å^−1^, which is the default in *CHROMIXS*) can enhance the protein signal and make it easier to select peaks in low signal-to-noise datasets, but makes it easy for the user to overlook issues outside that *q* range. Generally speaking, for a well behaved and reasonable signal dataset, either total integrated intensity or intensity in a specific *q* range (that covers a reasonable range from low to mid *q*) yield similar plots. Users may change the displayed value to see what works best for their dataset.

#### 4.1.1. New automated buffer- and sample-range selection

We have added the ability for *RAW* to automatically pick buffer and sample ranges from series datasets. This is aimed primarily at SEC-SAS datasets, but can be used for other series datasets with similar properties (*i.e.* a distinct constant buffer region and a distinct sample region). While this capability has been present in *CHROMIXS* (Panjkovich & Svergun, 2018 ▸) in the *ATSAS* suite for some time, we take a very different approach in *RAW*. For reference, the *CHROMIXS* approach automatically selects a sample range on the basis of peak-finding algorithms, often corresponding to the top of the strongest elution peak. Once the sample range is defined, a buffer range is selected by looking for contiguous low-intensity ranges of the same length as the selected sample range. A subtracted scattering profile is created using the buffer range, and the buffer range is then scored according to the quality of the Guinier fit. The best quality range is selected as the buffer.

The automated selection in *RAW* proceeds in the opposite order. First a buffer range is selected, and then a sample range is selected. The advantage to this order of operations is that additional information, such as trends in *R*_g_ or MW across an elution peak, can be incorporated to ensure that *RAW* picks an appropriate sample range in the dataset. The disadvantage to this order of operations is that *RAW* has less information for selecting the buffer range, so long low-intensity flat regions that contain an elution component can accidentally be selected as the buffer range.

**Figure 4:**
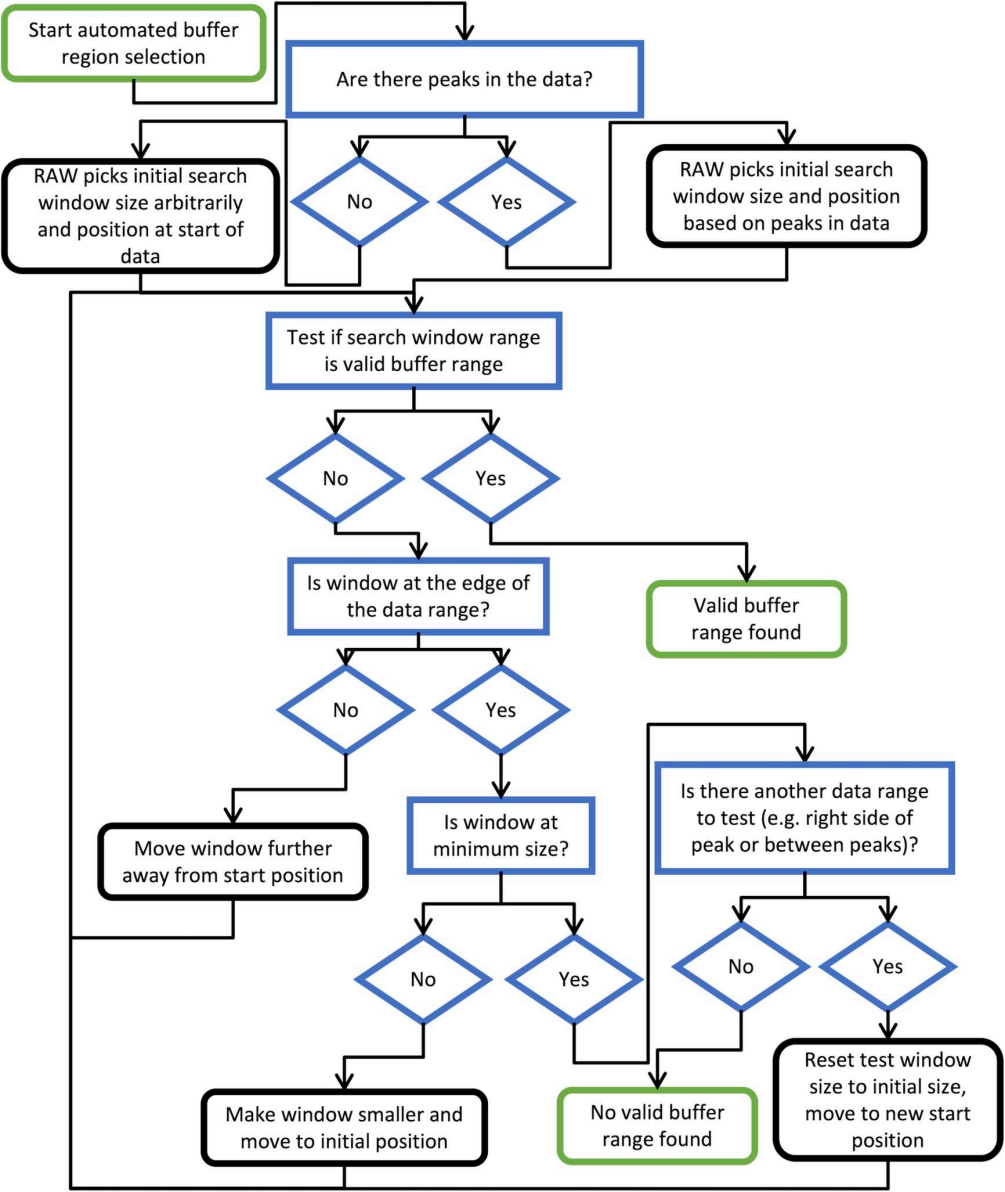
A flow chart for the automated buffer-range finding algorithm used by *RAW*. Green-edged boxes (rounded corners) are start and end points, blue-edged boxes (square corners) and diamonds are decision points or tests in the algorithm, and black-edged boxes (rounded corners) are actions by the algorithm.

*RAW*’s basic approach to finding a good buffer range is to scan a window of defined size along the measured profiles and test each range to see if it is a valid buffer range. If no valid range is found, the window size is narrowed and the scan repeated until either a valid range is found or the minimum size is reached. Additionally, *RAW* constrains the set of buffer ranges to test, both to avoid false positives and to improve the speed of the algorithm, on the basis of the elution peaks in the dataset. A schematic diagram of the search algorithm is given in Fig. 4 ▸. A more detailed look at the algorithm and the tests used to determine if a buffer range is valid is provided in Section S1 of the supporting information.

The test for a valid buffer range has two inputs. The first is the total intensity (or mean intensity or intensity in a given *q* range or at a particular *q* value, depending on user choice) versus frames (or time) data, sometimes called the scattergram, and the second comprises the scattering profiles at each measured point in the elution. *RAW* evaluates a buffer range in three ways. First, it tests for correlations in the total intensity. Buffer scattering should have the same intensity at all measured points, so correlations are indicative of something eluting in the data (or an issue with the baseline). For the second test, *RAW* checks the similarity of the scattering profiles in the test range. We expect all buffer profiles to be similar. *RAW* checks similarity across three different *q* ranges: the low *q* range, where we may see capillary fouling or damage; the high *q* range, where we may see baseline drift; and the full profile. In the third test, *RAW* checks the number of significant singular values in the scattering profiles in the range. Buffer ranges should only have one significant singular value, the buffer-scattering component. If any of these tests fail, the range is not a valid buffer range.

In order to optimize the speed of the automated buffer finding, *RAW* runs the parts of the test in the order listed above, from fastest to slowest, and if any part fails on the selected range, *RAW* does not run the subsequent parts.

*RAW* uses the same general approach for the automated sample-range determination as it does for the automated buffer-range finding. A window is scanned along the data and it tests whether each selected range is a valid sample range. If no valid range is found, the window size is narrowed and the scan repeated until either a valid range is found or the minimum size is reached. *RAW* again constrains the sample ranges to test to within the strongest elution peak, both to avoid false positives and to improve the speed of the algorithm. A valid sample range will not be determined if no peaks are found in the dataset. A schematic diagram of the search algorithm is given in Fig. S1 of the supporting information. A more detailed look at the algorithm and the tests used to determine if a sample range is valid is provided in Section S1.

The test for a valid sample range has three inputs: the scattering profiles and the *R*_g_ and MW values calculated for each profile in the selected range. There are five parts to the test. The first part is simply whether all profiles in the selected range have calculated *R*_g_ and MW values. If the selected range is good, these values should be calculated for all profiles in the range. The second part checks for correlations in the *R*_g_ and MW values in the range. If the sample is uniform across the selected range there should be no correlation. The third part tests for similarity between the subtracted scattering profiles in the selected range. We expect all subtracted profiles to be similar to within a scale factor. As with the buffer similarity test, the full *q* range, the low *q* range and the high *q* range are all tested. For the fourth part, *RAW* checks the number of significant singular values in the scattering profiles in the range. As with the buffer range, sample ranges should only have one significant singular value, the sample-scattering component. The fifth and final part of the test is to check whether including all the profiles in the selected range improves the signal to noise of the final averaged subtracted scattering profile. If including a profile in the average decreases the signal to noise, then that profile should not be included in the final dataset and so the selected range is not valid. If any of these tests fail, the range is not a valid sample range.

As with the automated buffer-range selection, in order to optimize the speed of the algorithm, *RAW* runs the tests in the order listed above, fastest to slowest, and if any test fails on the range, subsequent tests are not run.

Both the buffer- and sample-range finding algorithms in *RAW* are simply based on useful heuristics. There is no proven way of always finding valid sample and buffer ranges – this may not exist – so instead we have devised a set of metrics that corresponds to how a well trained human would evaluate the data (*e.g.* for the sample, it should have constant *R*_g_ and MW, the profiles should be the same, and we should not include data that are too noisy) and which yields results that consistently pass the eye test from users. The algorithms can yield invalid ranges, so the user should always examine and evaluate the automatically selected ranges before proceeding with further analysis, and modify them as necessary.

**Figure 5:**
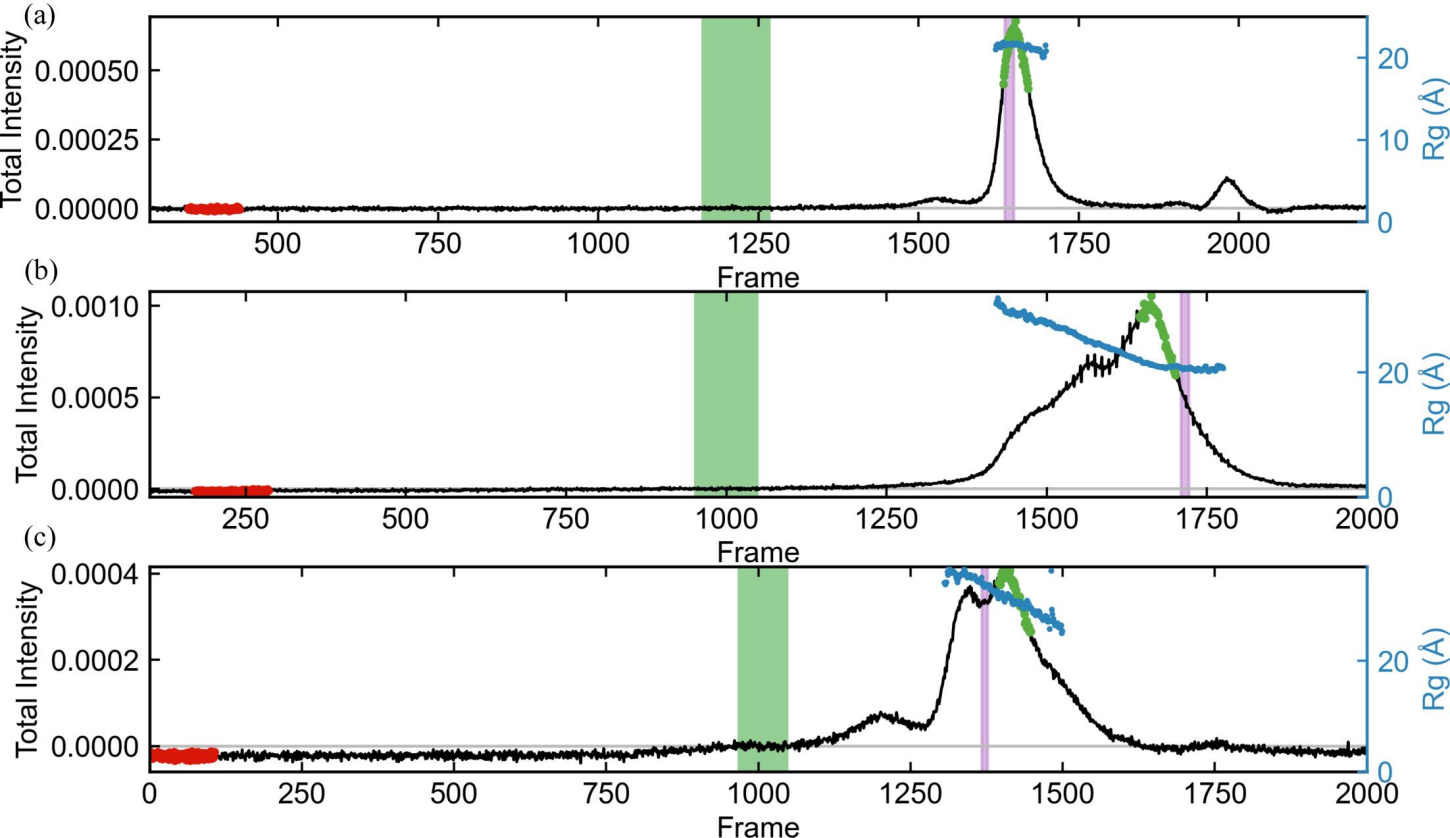
Automated buffer and sample regions as selected by *RAW* (green and purple shaded regions) and *CHROMIXS* (red and green dots) for various SEC-SAXS datasets. The total subtracted intensity for each dataset is shown in black (left axis) and the calculated *R*_g_ across the elution is shown in blue (right axis). Subtracted profiles were calculated using the buffer range selected by *RAW* and *R*_g_ calculations were carried out using *RAW*. The plots show the following. (*a*) Well behaved SEC-SAXS data showing a flat buffer region, a well separated single peak and relatively constant *R*_g_ across the peak. The sample ranges selected by *RAW* and *CHROMIXS* are overlapping, with *CHROMIXS* selecting a larger region while *RAW* excludes frames where the *R*_g_ starts to trend slightly downward on the edges of the peak. (*b*) A poorly separated sample showing only a small region of potentially usable constant *R*_g_ profiles on the right edge of the peak. Here, *RAW* selects frames in that constant *R*_g_ region, where *CHROMIXS* fails to do so and selects frames in the obviously overlapped region where *R*_g_ trends upwards. (*c*) A rare case where *RAW* selects a buffer range obviously within the elution region, resulting in incorrect background subtraction. *CHROMIXS* selects an appropriate buffer range.

We show a comparison of automatically determined buffer and sample ranges using *RAW* and *CHROMIXS* (from *ATSAS* 3.2.1) in Fig. 5 ▸. (Data for this figure are from proteins measured by users at the BioCAT beamline, used with permission, and the data are anonymized. A general data-collection protocol is given in Section S3.) For high-quality data [Fig. 5 ▸(*a*)], both programs yield similar, overlapping, sample ranges. Because the *R*_g_ calculated across the peak tails off slightly at the edges (starting near ∼21.3 Å on the left edge, plateauing near ∼21.6 Å in the middle and dropping to ∼20.8 Å on the right edge), *RAW* picks a more conservative range of the peak than *CHROMIXS* does, avoiding this tailing. There is a significant difference in selected buffer range, but as the buffer is uniform this results in no appreciable difference in the subtracted profile. In this case, the final profiles (not shown) are essentially identical, though the *CHROMIXS* one has slightly better signal to noise due to selecting more of the peak.

Several other datasets were selected to highlight where the algorithms can break down. In Fig. 5 ▸(*b*), the elution profile and sloping *R*_g_ clearly show that there are multiple overlapping components in the elution. Because it has access to the *R*_g_ information, *RAW* selects a sample range near the trailing edge of the peak where *R*_g_ is essentially constant. *CHROMIXS* selects a sample range covering a large portion of the peak where *R*_g_ varies. The resulting profiles (not shown) are not the same, and the profile from *RAW* is of a higher quality. While in cases like this we would recommend that users apply a deconvolution technique such as EFA rather than averaging ranges of the peak, the additional information available to *RAW* clearly generates a better result. We have not tested this kind of analysis extensively with *CHROMIXS*, so we cannot say how common an occurrence this inaccurate sample-range selection is when there are multiple components in solution.

Similarly, *RAW* is not immune to mistakes. In Fig. 5 ▸(*c*), *RAW* selects a buffer range that meets all the automated criteria (flat range, similar profiles, earlier than the first identified peak) but which is clearly in a region where protein is eluting. *CHROMIXS*, presumably because it includes the additional constraint of the best Guinier fit, avoids this and selects an earlier buffer range. (In this elution there is no good range to pick for the sample, as seen by the continuously sloping *R*_g_, but both algorithms return a result. The user should instead apply a deconvolution approach.) Again, we see a trade-off based on where one includes additional information. In our anecdotal (though extensive) experience, failures of this type are rare for *RAW*, and we believe the trade-off of this failure mode versus being able to determine what portions of the peak contain multiple components according to changes in the *R*_g_ and MW values is a valuable one.

#### 4.1.2. Testing and reporting on suitability of selected buffer and sample ranges

Whenever the user sets the buffer or sample range in the LC analysis panel, *RAW* validates the range using the tests described above. If any of the tests return an invalid result, a summary dialogue of the failed test results is shown to the user and the user is given the choice to continue with their selected range or to adjust the range. While it is often acceptable to include ranges with reported validation issues (in particular, for the sample range, there is often only a small range that is completely valid, so accepting some potential issues to improve the overall data quality can be a reasonable choice), the user is at least made aware of the issues and can decide for themselves if they want to proceed.

#### 4.1.3. Series baseline correction

We have added two baseline-correction algorithms to *RAW* to correct for drift or damage: a linear baseline correction that is useful for simple drift (such as from changes in temperature), first described by Brookes *et al.* (2013 ▸), and an integral baseline correction that is more useful to account for damaged species (sample or buffer) accumulating on the capillary (Brookes *et al.*, 2016 ▸). In both cases the user first subtracts the data and then defines start and end ranges for the correction. *RAW* averages the profiles in the start and end ranges to create the average start and end points used for the correction. The user can select whether the correction should be applied to just the data between the start and end points or all the data in the series. The linear correction is applied independently to each *q* value in a profile. In brief, for every *q* value, a simple linear fit in intensity between the start and end points is carried out, and then the intensity of that fit line at a particular profile is calculated and subtracted from that profile. The integral baseline correction uses the algorithm previously described and implemented in *US-SOMO* (Brookes *et al.*, 2016 ▸).

#### 4.1.4. New .hdf5 save format for series

Previously, *RAW* saved series data as a .sec file. While technically an open format, in that anyone could look up the specifications for reading or writing in the *RAW* code, it relied on the Pickle module in Python to serialize and compress the data, which meant that only other Python programs could open these files. We have changed *RAW* to use .hdf5 files to save series data, which is a more standard format that can be opened by any program with .hdf5 compatibility.

### 4.2. Improvements to automated Guinier fitting and *D*_max_ finding

The new version of *RAW* provides an updated heuristic algorithm for determining the range of a Guinier fit (‘auto Guinier’) and a new heuristic algorithm for finding the optimal maximum dimension (*D*_max_) for a *P*(*r*) function (‘auto *D*_max_’) being calculated with an IFT. In both cases, the algorithms were developed and tested against the data available in the Small-Angle Scattering Biological Data Bank (SASBDB; https://www.sasbdb.org) in fall 2020 (Kikhney *et al.*, 2020 ▸).

To demonstrate the utility of these automated methods, and to compare against other available methods, we compared the results against values determined by scientists from real experimental data. To this end, we ran the methods against all available biological macromolecular data in the SASBDB (as of June 2023). We used a simple Python script to scrape the SASBDB using the provided *REST* API, and downloaded the scattering profiles and the depositor-reported *R*_g_ and *D*_max_ values for all biological macromolecular entries. We only used entries with a molecule type listed in the SASBDB of protein, RNA or DNA, a total of 3138 datasets, treating the *R*_g_ and *D*_max_ values reported by the depositor/experimenter as the ‘true’ values for the dataset. We used the *RAW* auto Guinier and *D*_max_ algorithms from version 2.2.0 of *RAW*; *ATSAS* programs were from *ATSAS* version 3.2.1.

#### 4.2.1. Automated Guinier fitting

The basic implementation of the auto Guinier function was described previously (Hopkins *et al.*, 2017 ▸). We made two major changes in this updated version. First, some of the weighting coefficients on the different component tests were changed to yield better results according to tests against the SASBDB data. Second, the algorithm now undergoes a progressive relaxing of certain criteria and changing of the weights, which allows automatic determination of the Guinier range from lower-quality (noisy, aggregated, *etc.*) data. Here, *RAW* first does a relatively strict search, looking for only high-quality regions. If that fails to yield a suitable region, *RAW* allows a smaller window for the fit and lowers the minimum acceptable quality of the fit. If that fails, *RAW* then allows the fit to include more data, both by broadening the region of the profile over which the search takes place and by increasing the allowable range for minimum and maximum *qR*_g_ values. *RAW* also further reduces the quality threshold for a successful fit. These changes provide a much more robust function for low-quality data and yield high-quality results, as discussed below.

**Figure 6:**
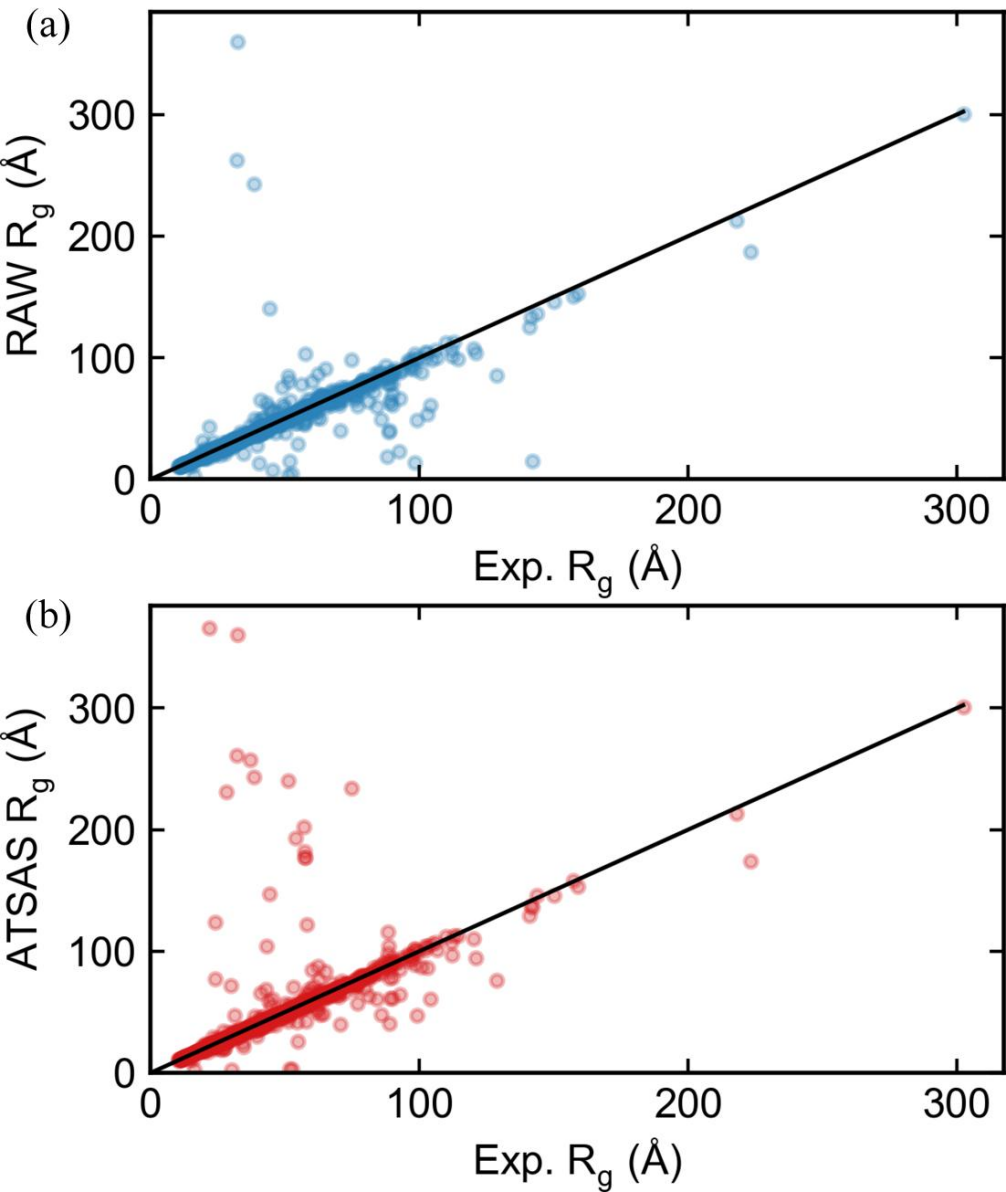
Plots of the *R*_g_ values automatically calculated by (*a*) the *RAW* automatic Guinier function and (*b*) the *ATSAS AUTORG* function on the *y* axis versus the experimenter-determined *R*_g_ values from a SASBDB entry on the *x* axis. Results are shown for all SASBDB entries with *R*_g_ values that were classified as either protein, DNA or RNA, unless the experimenter-determined Guinier range perfectly matched either automated method. Perfect agreement between the automated method and the experimental method would be equal *R*_g_ values, shown by the black line in each figure.

We ran the *RAW* auto Guinier algorithm and the *ATSAS AUTORG* program (Petoukhov *et al.*, 2007 ▸) on the collected SASBDB profiles to obtain Guinier fits. If the experimenter-reported starting and ending *q* values agreed precisely with either of the automated results, we did not include the dataset in subsequent evaluation, as the experimenter may have simply reported the value from an automated method and we wanted only to compare with manually determined values. We used the ratio of the automated values and the true *R*_g_ values to determine how close each automated determination was to the ‘true’ value. We also tracked the number of datasets where a method failed to return a value. Of the 3138 initial datasets, 1827 had values that did not match either of the automated methods tested and had experimenter-provided *R*_g_ values. From these, the average and standard deviation of the ratio of (experimenter-determined *R*_g_)/(automatically determined *R*_g_) was 1.04 ± 0.56 for the *RAW* auto Guinier method and 1.03 ± 0.63 for the *ATSAS AUTORG* method. Plots of the automated *R*_g_ versus experimenter-determined *R*_g_ values are shown in Fig. 6 ▸. *RAW* failed to return results for 5 (0.27%) datasets and *AUTORG* failed to return results for 11 (0.60%) datasets. Our results show that both algorithms are robust, in that they fail on less than 1% of all datasets, and that both algorithms are on average quite accurate. For comparison, the old *RAW* algorithm, run on the same set of data, was similarly accurate (*R*_g_ ratio: 1.02 ± 0.51) but failed on a significant number of datasets (181, 9.9%). When we used all datasets, including those where the experimenter-input *q* range matches that determined by one of the automatic methods, there was no significant change in the results (see Section S2.2).

#### 4.2.2. Automated *D*_max_ determination

The auto *D*_max_ function can run in several ways. If the *ATSAS* package is not available, it simply returns the *D*_max_ value found by BIFT. However, if the *ATSAS* package is available, then *D*_max_ can be fine-tuned to get a more accurate value. The basic idea is simple. First, *RAW* runs other automated methods – BIFT, *DATGNOM* (Petoukhov *et al.*, 2007 ▸) and *DATCLASS* – to determine a good starting point for the search. After determining a starting value, *RAW* calculates the *P*(*r*) function using *GNOM* with force to zero at the maximum dimension turned off. *RAW* checks this initial unconstrained *P*(*r*) function in two ways, first for overestimated *D*_max_ values and then for underestimated *D*_max_ values, and adjusts the maximum value until it finds a good *D*_max_.

In a *P*(*r*) function with an overestimated *D*_max_, we expect either a long tail oscillating about zero (for homogenous monodisperse non-interacting samples) or negative values near the maximum dimension (for data with repulsive interactions) (Jacques & Trewhella, 2010 ▸). Using these criteria, if *RAW* determines that *D*_max_ is overestimated, it decreases *D*_max_ in 1 Å increments and recalculates the unconstrained *P*(*r*) function using *GNOM* until these criteria are no longer satisfied. In a *P*(*r*) function with an underestimated *D*_max_, we expect that the value at the end of the *P*(*r*) function is significantly greater than zero (Jacques & Trewhella, 2010 ▸). Using these criteria, if *RAW* determines that *D*_max_ is underestimated, it increases *D*_max_ by 1 Å and recalculates the *P*(*r*) function until that is no longer the case. More details on how these criteria are applied to determine under- and overestimation are in Section S2.1.

*RAW* applies one additional constraint to the adjustments, constraining the change in *D*_max_ to be no more than 50%, either an increase or a decrease, of the initial value, to prevent the algorithm from running away. This is particularly useful in the cases of highly aggregated data where there may be no appropriate maximum dimension and the algorithm could otherwise increase *D*_max_ essentially indefinitely.

When taken together, these two simple adjustments for overestimated and underestimated *D*_max_ values provide a more robust estimate of the maximum dimension than any of the other tools mentioned above, though we rely on those tools to find an appropriate starting point and so our approach should be considered complementary to the previously developed methods.

**Figure 7:**
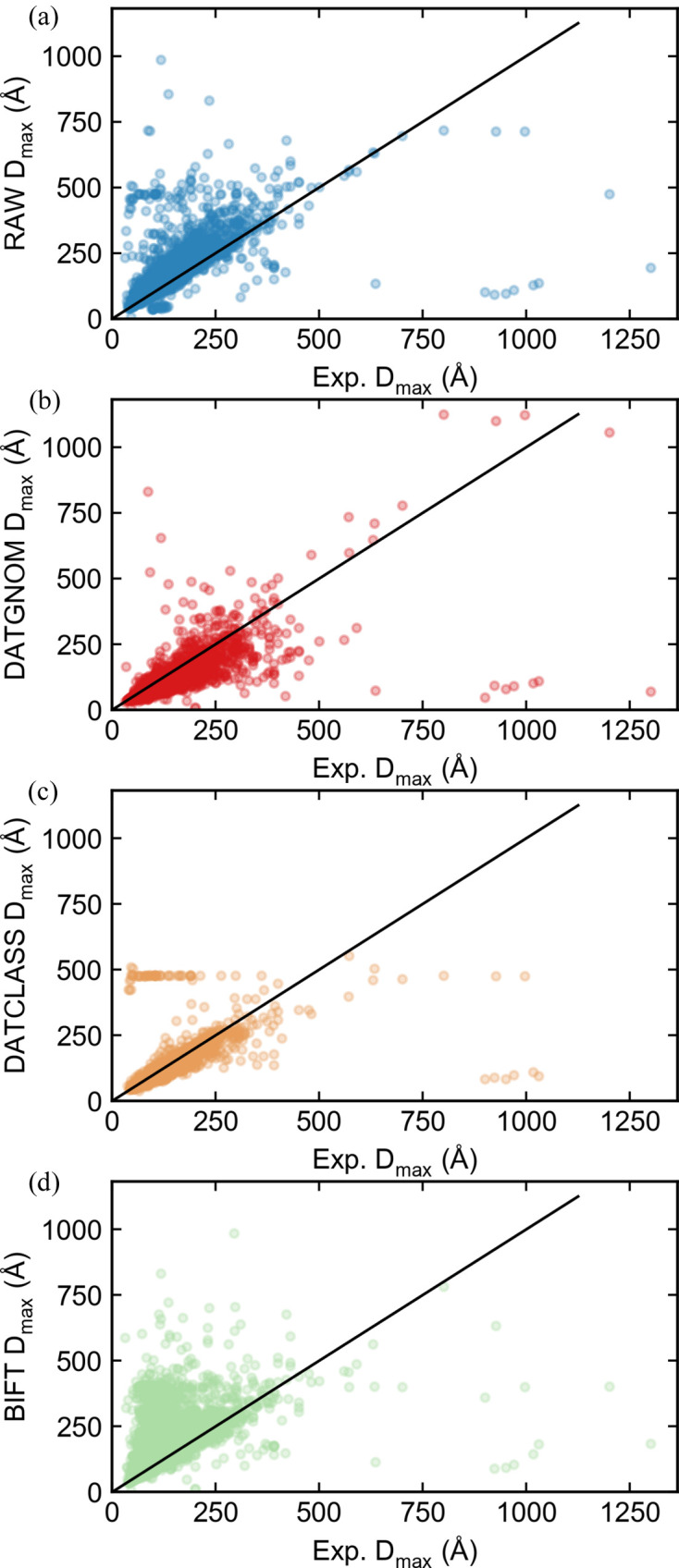
Plots of the automatically calculated *D*_max_ values by (*a*) the *RAW* auto *D*_max_ function, (*b*) the *ATSAS DATGNOM* function, (*c*) the *ATSAS DATCLASS* function and (*d*) BIFT (as implemented in *RAW*) on the *y* axis versus the experimenter-determined *D*_max_ values from a SASBDB entry on the *x* axis. Results are shown for all SASBDB entries with *D*_max_ values that were classified as either protein, DNA or RNA, unless the experimenter-determined *D*_max_ values perfectly matched any of the automated methods. Perfect agreement between the automated method and the experimental method would be equal *D*_max_ values, shown by the black line in each figure.

To test the accuracy of the automated *D*_max_ algorithms, we ran the *RAW* auto *D*_max_ algorithm, the BIFT function, and the *DATGNOM* and *DATCLASS* programs in the *ATSAS* package on the collected SASBDB profiles to obtain *D*_max_ values. As with the *R*_g_ tests, if the reported *D*_max_ values agreed precisely with any of the automated values (rounded to the integer precision reported in the SASBDB), we did not include the dataset in subsequent evaluation. We calculated the ratio of the experimenter-reported *D*_max_ values and the automated values to determine how close the automated methods were to the ‘true’ value. We also tracked the number of datasets where a method failed to return a value. Of the 3138 initial datasets, 2502 of them had values that did not match any automated method and had experimenter-provided *D*_max_ values. Table 1 ▸ shows the average and standard deviation of the ratio of (experimenter-determined *D*_max_)/(automatically determined *D*_max_) for the various methods, as well as the number of datasets where the algorithm failed to return a result. Plots of the automated *D*_max_ versus experimenter-determined *D*_max_ values are shown in Fig. 7 ▸. When we used all datasets, including those where the user-input *D*_max_ matches that determined by one of the automatic methods, there was no significant change in the results (see Section S2.3).

There are several interesting takeaways here. First, none of the automated *D*_max_ methods are as good as either of the automatic Guinier determination methods, so automatically determining *D*_max_ is still a challenge. This may well reflect the inherent uncertainties in the IFT method. Second, most of the methods tend to have a systemic bias, towards either underestimating or overestimating *D*_max_. In particular, *RAW* and BIFT both overestimate, *RAW* by about 11% and BIFT by about 15%, while *DATGNOM* tends to underestimate by about 25%. Of the non-*RAW* methods, *DATCLASS* is the closest on average to the experimental value. However, *DATCLASS* also fails for 20% of the datasets, while none of the other methods had any failures. Overall, *RAW* auto *D*_max_ is the best combination of robustness and accuracy, although this result is probably expected since we take the other three methods as an input and then refine.

### 4.3. Improved uncertainty estimation for Guinier fits

Uncertainty estimation in values derived from the Guinier fit, particularly the *R*_g_ and *I*(0) values, is challenging because there are two competing sources of uncertainty. First, there is the inherent uncertainty in carrying out the fit, which is easily captured by the covariance of the fit parameters (assuming accurate uncertainty estimates for the intensity values of the scattering profile). Second, there is the uncertainty in selecting the range of data to be fitted, which is not easy to quantify. We are certainly not the first to acknowledge this challenge; for example, in the *ATSAS* package, the uncertainty in the *R*_g_ and *I*(0) values from the *AUTORG* (Petoukhov *et al.*, 2007 ▸) function, which automatically determines the end points, is different (and typically larger) than the uncertainty provided using the *DATRG* function with the end points determined by *AUTORG*.

In order to provide more robust uncertainty values from the Guinier fit, we created an algorithm that estimates the effect of changing the end points of the Guinier range. *RAW* assumes that the end points should not be extended, *i.e.* that the user has picked the start and end points such that the data outside the selected range are not usable (for example, the start point may be picked to exclude aggregation or repulsive effects, while the end point is typically picked at the largest *q* value where the Guinier approximation still holds). *RAW* generates a series of sub-ranges contained within the user-selected Guinier range. It then calculates the *R*_g_ and *I*(0) values for Guinier fits to these sub-ranges. *RAW* takes the standard deviation of these values and provides that as an additional uncertainty estimate, the range-induced uncertainty. *RAW* shows a top-level uncertainty value in the Guinier fit window that is the greater of either the fit uncertainty or the range uncertainty, and the individual values are also provided so the user can decide which they wish to use.

For well behaved data and well selected Guinier ranges, the largest source of uncertainty is typically from the covariance of the fit parameters. However, for data that display systematic non-linearities (due to the presence of aggregation, repulsion or a poorly selected fit range that extends past a *q* range where the Guinier approximation is valid), the range uncertainty is often the largest uncertainty. For example, using data from the *RAW* tutorial, the provided glucose isomerase data are high quality and return an *R*_g_ of 33.89 Å over the range determined by *RAW*’s automated Guinier fit function. For these data, the fit uncertainty is 0.24 Å and the range uncertainty is 0.10 Å. In this case, the data all fall on the expected Guinier fit line, so the uncertainty from the measured intensity, which results in the fit uncertainty, is larger than that of the choice of fit end point. The tutorial data also include highly repulsive data from concentrated lysozyme. The reported *R*_g_ here is 12.18 Å, with a fit uncertainty of 0.015 Å and a range uncertainty of 0.18 Å, indicating that because there is a repulsive effect the choice of range introduces much more variation in the Guinier fit values than the uncertainty in the measured intensity.

If the Guinier range is determined using *RAW*’s automated method, instead of showing the range uncertainty described above, *RAW* provides a similar value, the standard deviation of the *R*_g_ and *I*(0) values of all ranges found during the automated search that exceed a specified quality threshold (analogous to the uncertainty provided by the *ATSAS AUTORG* function).

### 4.4. Handling of multi-image files

Many modern detectors, such as the commonly used EIGER (Dectris) detectors, use file formats that package multiple images into a single file (*e.g.* an .hdf5 file). We have updated *RAW* to be able to efficiently load in and radially average multi-image files, including the ability to determine the appropriate file numbering for series data loaded from several multi-image files, as well as display images from these files. The FabIO Python library (Knudsen *et al.*, 2013 ▸) provides the basic reading of the file formats, while *RAW* deals with which parts of the file to process or display.

### 4.5. Use of *pyFAI* for radial averaging

Since the last report about *RAW*, we have modified *RAW* to use the *pyFAI* Python library (Ashiotis *et al.*, 2015 ▸; Kieffer *et al.*, 2020 ▸) for radial averaging, as well as detector calibration. The major advantage of this change is that *pyFAI* is extremely fast and can utilize GPUs, so that even very large detector images can be processed quickly. *pyFAI* also incorporates additional corrections not available in the previous *RAW* radial-averaging algorithm, such as correcting for incident beam polarization direction. This change has allowed *RAW*’s radial averaging to keep up with the demands of modern data collection, in terms of both image size and frame rate.

### 4.6. *ATSAS* integration

*RAW* provides an interface for several programs from the *ATSAS* package (separate installation required) (Manalastas-Cantos *et al.*, 2021 ▸) including *AMBIMETER* (Petoukhov & Svergun, 2015 ▸), *CIFSUP* (*ATSAS* version 3.1.0 or newer), *CRYSOL* (Svergun *et al.*, 1995 ▸; Franke *et al.*, 2017 ▸), the Bayesian inference MW method in *DATMW* (Hajizadeh *et al.*, 2018 ▸), *DAMMIF* (Franke & Svergun, 2009 ▸), *DAMMIN* (Svergun, 1999 ▸), *DAMAVER* (Volkov & Svergun, 2003 ▸), *DAMCLUST* (prior to *ATSAS* version 3.1.0) (Petoukhov *et al.*, 2012 ▸), *DATCLASS* (Franke *et al.*, 2018 ▸), *GNOM* (Svergun, 1992 ▸), *SASRES* (Tuukkanen *et al.*, 2016 ▸) and *SUPCOMB* (prior to *ATSAS* version 3.1.0) (Kozin & Svergun, 2001 ▸).

Many of these programs are newly supported since the last publication about *RAW*. Users can now use *RAW* to align high-resolution models with bead models via *SUPCOMB/CIFSUP* (depending on the *ATSAS* version). They can do this either automatically when generating bead models as a final step in the process or through the alignment window with any two saved models. Additionally, *RAW* now allows users to calculate theoretical scattering profiles from high-resolution structures/models (either .pdb or .cif formats) using *CRYSOL*. If a user simply loads in a model the calculation is done automatically, or they can use the *CRYSOL* window to adjust the settings in *CRYSOL*, fit the model to measured experimental scattering data, and see a plot of the theoretical profiles and, if applicable, the normalized residuals to the experimental data. We have also added support for the new MW methods using Bayesian inference and machine learning, from the *DATMW* and *DATCLASS* programs. These are available in the MW window.

*RAW* is only tested against the latest version of *ATSAS*, so compatibility is only guaranteed for that version. However, attempts are being made to preserve backwards compatibility, so previous versions will usually work; for example, limited testing suggests that *RAW* 2.2.1 is compatible with *ATSAS* back to versions 3.0.*X* (which were 2–3 years old when this was written), as well as being fully compatible with 3.2.1 (the current latest release). If backward-compatibility breaking changes are made to the *ATSAS* programs between releases, *RAW* will not be compatible with the most recent *ATSAS* release until a new version of *RAW* is subsequently released.

### 4.7. Improvements to BIFT to add Monte Carlo error estimation and extrapolation of regularized curve to *I*(0)

We have updated the implementation of the BIFT method (Hansen, 2000 ▸) in *RAW* to provide Monte Carlo error estimation for the generated *P*(*r*) function and to extrapolate the regularized scattering profile to *I*(0). These updates coincided with moving the BIFT code from the depreciated scipy.weave compiler to the *Numba* just-in-time (JIT) compiler.

### 4.8. New documentation

For prior versions of *RAW*, we created the documentation manually in a Word document, saved it as a PDF and uploaded it to the website with each new release of *RAW*. This was labour intensive, hard to update quickly and awkward to actually use. As part of a complete overhaul of the documentation, we now create the documentation using the *Sphinx* Python package and host it on Read the Docs. This allows us to put the documentation in the program’s git repository, meaning it is now version controlled. With *Sphinx* it is easy to build multiple different formats (including the html version on the web, and downloadable PDF, html and epub versions), and updating it simply requires initiating a rebuild on Read the Docs, which grabs the latest version from the git. Read the Docs also provides multiple versions simultaneously. The latest version is the default, but every version of the documentation from the latest back to 1.3.1 is available (documentation versioning matches that of the *RAW* releases). The documentation is also distributed in html format with the *RAW* software, and is accessible on Windows and macOS systems via the in-program ‘Help’ menu.

We use the autodoc function in *Sphinx* to automatically generate the API documentation for *RAW*, using the docstrings written as part of the API. In addition to tutorials on how to use the program itself, the website has tutorials on best practices for basic SAXS operations, like performing Guinier fits, calculation of MW, generating *P*(*r*) functions via IFTs and making bead-model reconstructions (https://bioxtas-raw.readthedocs.io/en/latest/saxs_tutorial.html).

In addition to the written documentation, most tutorials are now accompanied by YouTube videos (https://youtube.com/playlist?list=PLm39Taum4df4alFnacOOr1RWgylwiTWED), 23 in total with roughly 3.5 h of runtime. These videos provide a useful alternative to the written tutorials for those who learn best by seeing the program in action. Finally, there is also a new (since the last report about *RAW*) mailing list where announcements about new versions are made and users can report bugs or ask questions (https://groups.google.com/g/bioxtas_raw).

## 5. New features

### 5.1. Comparison window

Since the previous *RAW* publication, we have added a dedicated window for easy comparison of multiple scattering profiles. The comparison window allows the user to compare profiles using the residual between profiles, the ratio of the profiles or a statistical test [currently the CorMap (Franke *et al.*, 2015 ▸) test implemented in *RAW*]. For both residual and ratio comparisons, the selected profiles are compared against a user-selected reference profile. The user is shown a two-panel plot with all of the selected profiles plotted on the top and the appropriate metric plotted on the bottom. The user may then toggle profiles on or off, change which profile is the reference profile used for comparison, change the scaling of the plot axes, scale the profiles to the reference profile and, for the residuals plot, display uncertainty-normalized or unnormalized residuals. For the similarity test, each selected profile is compared against all the other selected profiles. The results are shown as a heatmap plot of the *p* value from the test, and in a list that gives the pair of compared profiles and the test result and *p* value. The results of the similarity test can be exported to a comma separated value (csv) file.

### 5.2. PDF reports

A major new feature in *RAW* is the ability to save a summary of your data processing for individual profiles, *P*(*r*) functions and series data as a PDF report. A summary of *DAMMIF/N* and *DENSS* results produced by *RAW* can also be included. The report provides summary plots and tables of the key results, along with detailed tables of experimental parameters (when available) and all of the analysis results. The report is flexible, in that any or all of the data types can be saved in the same report, and more than one of each can be saved, in which case results are plotted on the same plots and added as additional columns to tables. Plots are saved as vector graphics and so are suitable for inclusion in a publication (anecdotally, a number of these summary plots have been seen ‘in the wild’, usually in supporting information in lieu of the authors making their own versions). The window for generating the report makes it easy for the user to select which datasets loaded into *RAW* are included in the report by simply checking and unchecking boxes.

The summary plots saved in the report include a plot of series intensity versus frame number and *R*_g_ versus frame number with selected buffer and sample ranges indicated, a log–lin plot, a Guinier plot with fit line and residual, a dimensionless Kratky plot for profiles, and an *I*(0)-normalized *P*(*r*) plot for *P*(*r*) functions. If the user does deconvolution on a series with EFA or *REGALS* then summary deconvolution plots are also saved. The summary table includes top line information such as *R*_g_ and *I*(0) values, MW values, and *D*_max_ values. Analysis-specific tables provide more details; for example, the Guinier analysis table provides the *q* range used for the Guinier fit, the *q*_min_ *R*_g_ and *q*_max_ *R*_g_ values, and the *r* ^2^ of the fit, in addition to the *R*_g_ and *I*(0) values. A table of experimental parameters, such as date, experiment type, sample and buffer information, temperature, loaded volume and concentration, detector used, wavelength *etc*. is generated when these values are known. This typically requires that the reduction from images to one-dimensional profiles be carried out in *RAW* and that the beamline includes specific keywords in its metadata that *RAW* can recognize.

### 5.3. REGALS

*REGALS* is a new method for deconvolving mixtures from SAS datasets (Meisburger *et al.*, 2021 ▸). It can be applied to deconvolving overlapping peaks in SEC-SAXS, changing baseline and elution peaks in IEC-SAXS, mixtures in time-resolved SAXS data and equilibrium titration series data, and probably other cases that we have not explored. We have created a GUI for *REGALS* in *RAW* that allows for fast interactive deconvolution and provides initial singular value decomposition (SVD) analysis of the entire dataset, evolving factor plots and SVD analysis of the background outside of the peak range (for chromatography data) to help the user determine how many components to use and where to start and end each range. Because the *REGALS* code is open source, *REGALS* is distributed with *RAW* and no additional installation is necessary for users (unlike the *ATSAS* programs), and changes based on the incorporation of *REGALS* into *RAW* have been pushed back to the original *REGALS* codebase.

Though the mathematics are not the same, from a practical point of view it can be thought of as an extension of the previously implemented EFA technique (Meisburger *et al.*, 2016 ▸) to more complex conditions where components are not necessarily entering and exiting the dataset in a strict first-in/first-out approach like in SEC-SAXS. We still generally recommend EFA for standard SEC-SAXS data due to ease of use, but for more complex data as listed above, *REGALS* is preferred. *REGALS* can also handle deconvolution of SEC-SAXS data with a sloping baseline, something that EFA tends to fail at.

### 5.4. DENSS

Relatively recently, a new algorithm for determining actual (low-resolution) electron density from solution scattering data (*DENSS*) was developed (Grant, 2018 ▸). This provides an alternative to the more traditional bead models. *RAW* provides users with a simple user-friendly GUI for running *DENSS*, including creation of a number of individual density maps, averaging and refinement to create a final map, alignment of high-resolution structural models to the final density map, and evaluation of the results. The interface is very similar to the GUI for making bead models in *RAW*, making it easy for users to try either or both approaches for their data. Because the *DENSS* code is open source, *DENSS* is distributed with *RAW* and no additional installation is necessary for users (unlike the *ATSAS* programs), and changes based on the incorporation of *DENSS* into *RAW* have been pushed back to the original *DENSS* codebase.

## 6. The *RAW* API

A new capability for *RAW* is the ability to use it via an API mode without the associated GUI. In this case, the user installs *RAW* as a standard Python package and then imports it into a standalone Python script, interactive terminal session (*e.g.* *IPython*), *Jupyter Notebook* or another Python program. The API provides access to all of the data reduction and analysis tools and algorithms available in the GUI, as well as direct access to the underlying experimental data objects that can be manipulated and saved. Profiles, *P*(*r*) functions and series generated in the GUI can be loaded by the API, and *vice versa* for complete cross compatibility. The API is fully documented and several usage examples are provided as part of the *RAW* documentation (https://bioxtas-raw.readthedocs.io/en/latest/api.html).

Having a headless version of *RAW* has several significant use cases. The first is that it can now be used to process data in a much more automated fashion than the GUI allows, something that we have taken advantage of in the beamline processing pipeline at the BioCAT beamline (https://github.com/biocatiit/saxs-pipeline). The second is that it can be used to create custom processing scripts for data that cannot be easily handled in the GUI: for example, in cases where the number of profiles makes manual processing prohibitive or where the dataset is not structured in a way that the GUI can handle. One example of this that fits both criteria is the time-resolved SAXS dataset used by Martin, Harmon *et al.* (2021 ▸). The initial dataset involved ∼220 000 images (across all the conditions and repeats), which were reduced, averaged and analysed initially by custom scripts using the API to yield the several hundred final profiles (over several different time series) that were then analysed by a combination of automated and manual approaches. The reduction and initial analysis scripts using the API are available as part of the supporting information of the referenced work (the scripts were written for an older version of the API and may not run in the current version without modification).

## 7. Technical updates

We have made some significant technical changes to the *RAW* program since the previous publication. The most important of these was the update to Python 3 (Python Software Foundation, https://www.python.org/) compatibility and the replacement of the depreciated scipy.weave compiler with the *Numba* JIT compiler for code that needed significant speed improvements. Both of these updates were critical for the ongoing maintenance and development of *RAW* and the ease of distribution of pre-packaged versions. We routinely update *RAW* to maintain compatibility with the latest version of the Python packages used by the program. As *RAW* also provides a GUI for various programs in the *ATSAS* package (requires separate installation) (Manalastas-Cantos *et al.*, 2021 ▸), we also carry out routine maintenance to retain compatibility with new versions of *ATSAS*. Additionally, we moved *RAW* from an svn to a git for version control of the code and migrated the code from Sourceforge (still used to host release files) to GitHub.

One additional advantage of the creation of the *RAW* API is that it became possible to use *pytest* to create a large test suite with relatively comprehensive coverage for all non-GUI functionality in *RAW* (*i.e.* an analysis function can be automatically tested but the GUI window that runs the analysis has to be manually tested). This is important to ensure functionality remains the same between versions and only intended changes occur. We test *RAW* on Windows, macOS and Linux before each release.

As of this publication, the explicit package dependencies of *RAW* are *Cython* (https://cython.org/), *dbus-python* (Linux only) (https://dbus.freedesktop.org/doc/dbus-python/), *fabio* (Knudsen *et al.*, 2013 ▸), *future* (https://python-future.org/), *h5py* (https://www.h5py.org/), *hdf5plugin* (https://www.silx.org/doc/hdf5plugin/latest/), *matplotlib* (Hunter, 2007 ▸), *mmcif_pdbx* (https://mmcif-pdbx.readthedocs.io/en/latest/), *Numba* (Lam *et al.*, 2015 ▸), *numpy* (Harris *et al.*, 2020 ▸), *pillow* (https://pillow.readthedocs.io/en/stable/index.html), *pyFAI* (Ashiotis *et al.*, 2015 ▸; Kieffer *et al.*, 2020 ▸), *ReportLab* (https://www.reportlab.com/), *scipy* (Virtanen *et al.*, 2020 ▸), *six* (https://six.readthedocs.io/), *svglib* (https://github.com/deeplook/svglib) and *wxPython* (https://wxpython.org/). Additionally, we use *Sphinx* (https://www.sphinx-doc.org/en/master/) and the *sphinx_rtd_theme* (https://github.com/readthedocs/sphinx_rtd_theme) to build the documentation, *pytest* (https://github.com/pytest-dev/pytest) for testing, and *PyInstaller* (https://pyinstaller.org/) to make the pre-built binaries. Most of these packages in turn depend on many other packages in the Python ecosystem.

## 8. Conclusions

*RAW* is a free open-source program that provides a wide range of basic and advanced analysis techniques for small-angle scattering. Recent improvements include significantly expanded series processing capabilities, new automated methods for Guinier fitting and *D*_max_ determination, the ability to perform electron-density reconstructions, generation of PDF reports, a new API, and migration to Python 3. Designed to be an easy-to-learn program that can carry users from images through subtracted scattering profiles, model-free analysis and some basic model-based analyses, the *RAW* package has, we believe, a vital place in the SAS data analysis ecosystem and will continue to prove useful for users for years to come.

## 9. Related literature

The following references are only cited in the supporting information for this article: Kirby *et al.* (2016 ▸).

## Supplementary Material

Supporting information. DOI: 10.1107/S1600576723011019/jl5075sup1.pdf

## Figures and Tables

**Table 1:** The average and standard deviation of the ratio of (experimenter *D*_max_)/(auto *D*_max_) for different automated *D*_max_ determination methods used on the biological data in the SASBDB, and the number of failures for each method

Algorithm	Mean ratio ± standard deviation	Number of failures
*RAW* auto *D*_max_	0.89 ± 0.51	0 (0%)
BIFT	0.84 ± 0.69	0 (0%)
*DATGNOM*	1.27 ± 0.98	0 (0%)
*DATCLASS*	1.06 ± 0.56	501 (20.0%)
